# A fish fry dataset for stocking density control and health assessment based on computer vision

**DOI:** 10.1016/j.dib.2024.111075

**Published:** 2024-10-28

**Authors:** Yuqiang Wu, Huanliang Xu, Bowen Liao, Jia Nie, Chengxi Xu, Ziao Zhang, Zhaoyu Zhai

**Affiliations:** a College of Artificial Intelligence, Nanjing Agricultural University, Nanjing 210095, Jiangsu, China; b College of Information Technology, Nanjing Police University, Nanjing 210023, Jiangsu, China

**Keywords:** Industrial aquaculture, Fry counting, Small target detection, Density control, Health assessment

## Abstract

Fish farming is a promising economic activity that promotes the social development, protects the ecological environment, and enhances the quality of human life. In recent years, various computer vision models have been established for assessing aquaculture density and monitoring fish health. However, existing datasets are generally characterised by larger fish sizes and low density, making them unsuitable for detecting small targets such as fish fry. This paper presents a dataset comprising 1101 images of largemouth bass (*Micropterus salmoides*) fry, specifically designed for small target detection in dense scenes. Each image contains a variable number of fish fries, ranging from 20 to 80 individuals. To facilitate health assessment in the aquaculture, a small number of dead fish fries are included in each image. The entire dataset is annotated with a total of 51,119 live fish fry and 3586 dead ones. Additionally, among the 80 images depicting high-density scenarios, there are complex situations such as overlap, occlusion, and adhesion, which pose challenges to the small target detection task. The dataset is annotated using the Labelimg tool and converted to the COCO format. It can be applied to a variety of scenarios, including seedling rearing, fry retailing, and survival assessments. It is also valuable for biomass estimation and aquaculture density control applications. In summary, this dataset provides an invaluable resource for the research community, advancing studies on fry counting and fish population health, thus contributing to the development of intelligent aquaculture.

Specifications Table

**Table:** 

Subject	Computer Vision, Object Detection, Aquaculture
Specific subject area	A dataset that helps detect small targets under dense scenes, specifically designed for fish fries.
Type of data	Image and corresponding annotation files.
Data collection	At a fish farm in Pukou District of Nanjing, Jiangsu Province, China, we employed a digital single-lens reflex camera to capture video footage of fish fry under four different illumination conditions and stocking densities. Each video was recorded under controlled lighting to ensure consistent data quality. A total of 1101 images were obtained by extracting frames from the videos and stored in JPEG format with a uniform resolution of 1080 × 1920. The dataset contains anchor annotations in both YOLO and JSON formats. Each image features both live and dead fish fry, resulting in 54,705 annotated bounding boxes across the entire dataset, comprising 51,119 live fish and 3586 dead fish.
Data source location	BlueVision Team, College of Artificial Intelligence, Nanjing Agricultural University.
Data accessibility	Repository name: Mendeley Data Data identification number: DOI: 10.17632/y52ffd3xdc.1 Direct URL to data: https://data.mendeley.com/datasets/y52ffd3xdc/1
Related research article	Xu, H., Chen, X., Wu, Y. et al. (2024). Using channel pruning–based YOLOv5 deep learning algorithm for accurately counting fish fry in real time. Aquacult Int. 10.1007/s10499-024-01609-x.

## 1 Value of the Data

• The dataset facilitates the accurate counting of fish fries, which is essential for optimizing stocking density and maintaining ideal conditions for healthy fish growth. Automated counting reduces the labor costs associated with manual efforts, increases the efficiency of aquaculture process, and provides data to support dynamic adjustment of aquaculture strategies.
• Regular monitoring the number and behavior of fish fries using the dataset allows for early detection of health problems, enabling preventive measures can be taken. This early warning system helps minimize losses, improve survival rates and promote overall fish welfare.
• The images in the dataset present a variety of complex scene challenges, such as dense small targets, overlap, occlusion, and adhesion, closely mimicking real-world aquaculture environments. This provides a rich set of training and validation resources for developing and optimizing deep learning-based visual models. Besides evaluating and comparing the performance of different models, it will also assist researchers in refining algorithms to enhance counting accuracy, especially when dealing with small targets in dense scenarios. The dataset advances the application of computer vision technology in aquaculture and lays the groundwork for future technological advancements.

## 2 Background

Computer vision has emerged as a transformative technology within aquaculture [1]. Its applications range from automated fish counting to behaviour analysis, providing valuable insights into the health and well-being of aquatic species [[2], [3], [4]]. However, the utilization of deep learning algorithms for these tasks relies on the availability of robust datasets. Despite the growing interest in applying computer vision to aquaculture, there remains a relative scarcity of data related to critical aspects such as fry density management and health assessment. This lack of datasets impedes the development and refinement of deep learning models tailored to the unique challenges of aquatic environments. To address this gap, we have created a specialized dataset featuring 1101 annotated images of fish fry across four stocking densities. This dataset will serve as a foundational resource for developing sophisticated computer vision applications within aquaculture, enabling the training of deep learning models that can accurately assess fish density and evaluate fish population health, thereby contributing to the advancement of smart aquaculture technologies. The dataset is poised to significantly impact the field of computer vision, particularly in agricultural and biological applications. It will empower researchers and practitioners to develop algorithms that are not only accurate but also adaptable to the complexities of real-world aquaculture settings. Furthermore, this dataset will foster innovation by providing a standardized benchmark for evaluating the performance of computer vision systems in dense and challenging environments.

## 3 Data Description

**Fig. 1:**
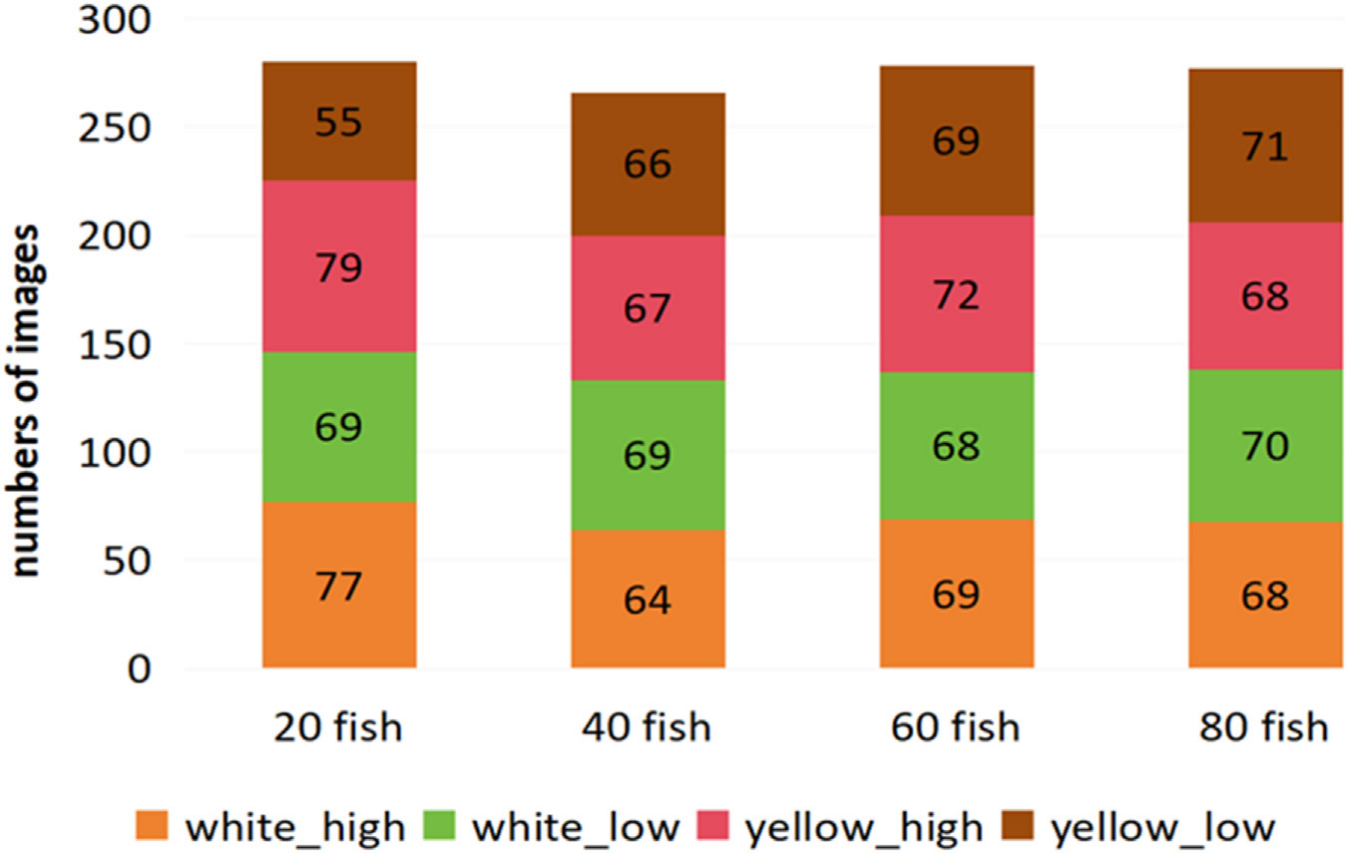
The number of images of fish fry under different density scenes.

Motivated by the importance and necessity of benchmarks like ImageNet [5], Pascal VOC [6], and MS COCO [7], this paper presents an image dataset of fish fries, called fish fry dataset (FFD), for breeding density control and health assessment. The fish images were extracted from video files with a resolution of 1080 × 1920. FFD comprises a total of 1101 images, yielding sufficient data to train a deep learning network. The maximum number of fishes in the box is 81, while the minimum number is 18, and the average is 50. Fig. 1 depicts a bar chart illustrating the quantities of fish across different farming densities under varying light conditions. The dataset possesses the following characteristics (1) fry images under four different conditions: white light at high water level (white_high), white light at low water level (white_low), yellow light at high water level (yellow_high), and yellow light at low water level (yellow_low); (2) the images contain varying density distributions; (3) the images include both live and dead fish fry; (4) overlapping, occlusion, and adhesion situations are present in the images; and (5) images are annotated in both YOLO and COCO formats. These features introduce new challenges for object detection algorithms that may perform well on conventional Pascal VOC and MS COCO datasets. We hope this dataset can be utilized in diverse ways to improve the performance of multi-target fish fry detection for precision fisheries breeding and benefit the deep learning community in algorithm evaluation.

The dataset is archived in a compressed file designated as FFD.zip, which is openly available to the public via url (https://data.mendeley.com/datasets/y52ffd3xdc/1 [8]). Upon decompression of this file, a directory labeled “Fish Fry Dataset” is generated. This folder contains four subdirectories: annotations, test, train, and validation. The annotations folder includes label files for the training set (instances_train.json), validation data (instances_val.json), and test set (instances_test.json). The train, validation, and test directories house images along with their corresponding YOLO-format labels, organized in a 6:2:2 ratio. Users have the flexibility to adjust these data proportions according to their specific requirements for training, validation, and tesing. The naming convention for the image and YOLO label files is structured as follows:  

JPEG images: X_Y_Zdead_W.jpg

**Fig. 2:**
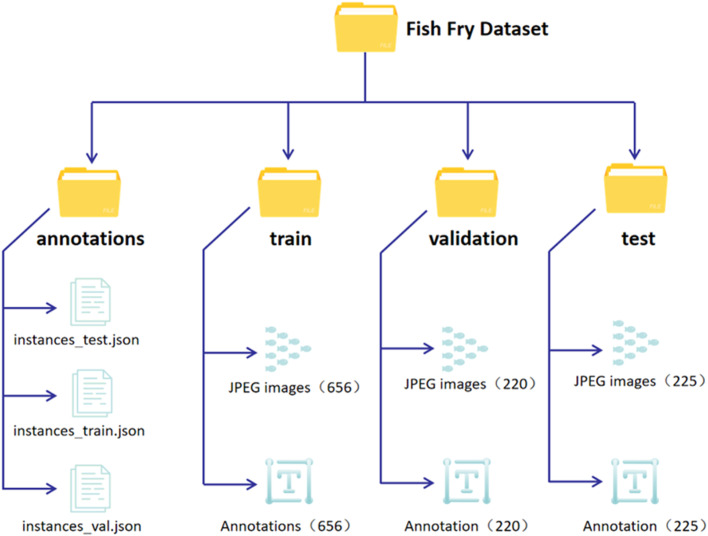
Organization of the dataset.

Annotations: X_Y_Zdead_W.txt  

where X represents stocking density, Y denotes lighting and water level conditions, Z indicates the number of dead fish fries, and W signifies the sequential number of the image within the dataset split at that stocking density. Fig. 2 provides a visual representation of the dataset's folder structure.

## 4 Experimental Design, Materials and Methods

### 4.1 Data collection experimental design

**Fig. 3:**
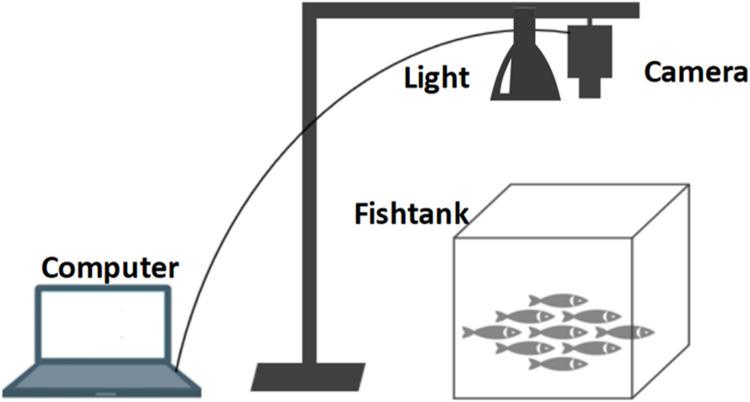
The structure of the experimental system.

This section outlines the methodologies employed to generate the data. At a fish farm located in Pukou District of Nanjing, Jiangsu Province, an image acquisition system was established, comprising a camera, a variable LED light source, a 50 cm × 50 cm square fish tank made of white polyethylene, and a computer. The camera used was a Cannon EOS 7D, which records videos of fish fry from a distance of approximately 50 cm above the water surface of the tank. The light source was capable of emitting both white and yellow light. During the data collection phase, fry videos were captured at low water depth of 10 cm and high water depth of 20 cm under the two different lighting conditions, resulting in four categories of videos: white_high, white_low, yellow_high, and yellow_low, each corresponding to different aquaculture densities. A schematic diagram of the experimental setup and data collection is shown in Fig. 3.

**Fig. 4:**
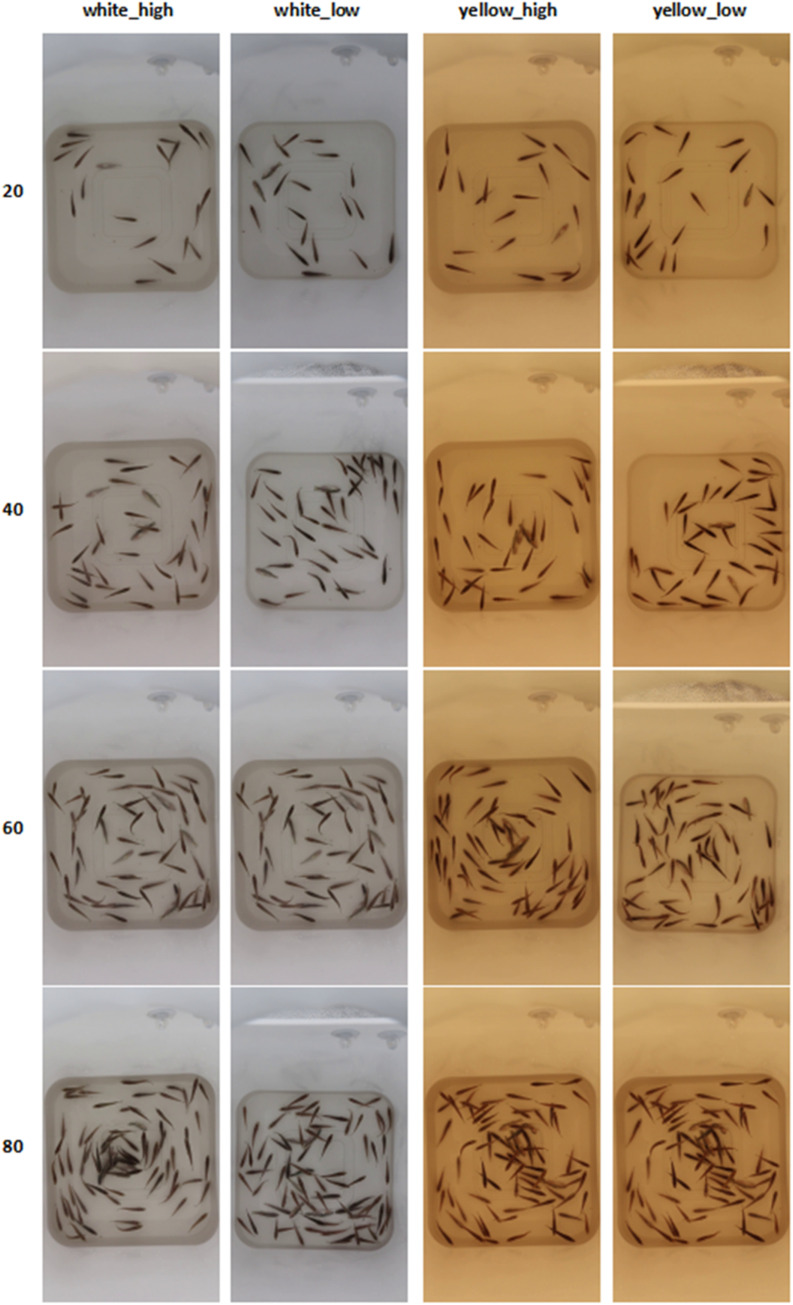
Examples of dataset images under different conditions.

Data collection focused on largemouth bass with body lengths ranging from 3 to 5 cm at four distinct stocking densities of approximately 20, 40, 60, and 80 fish. The fish fries were purchased from an authenticated fish farm (Nanjing Aquatic Breeding Center, Nanjing, Jiangsu, China) and the species was confirmed as the largemouth bass (*Micropterus salmoides*) by a professor from the College of Animal Science & Technology, Nanjing Agricultural University. Videos were recorded, showcasing aggregated, dispersed and swimming scenes under the aforementioned conditions. Each video session lasted approximately two minutes, after which the recorded videos were transferred to a computer for storage. Subsequently, one frame was extracted from the video every 0.5 s, resulting in a dataset of 1101 sea bass images, each with a resolution of 1080 × 1920 pixels. Fig. 4 illustrates the dataset images captured under four different density, lighting, and water depth conditions.

**Fig. 5:**
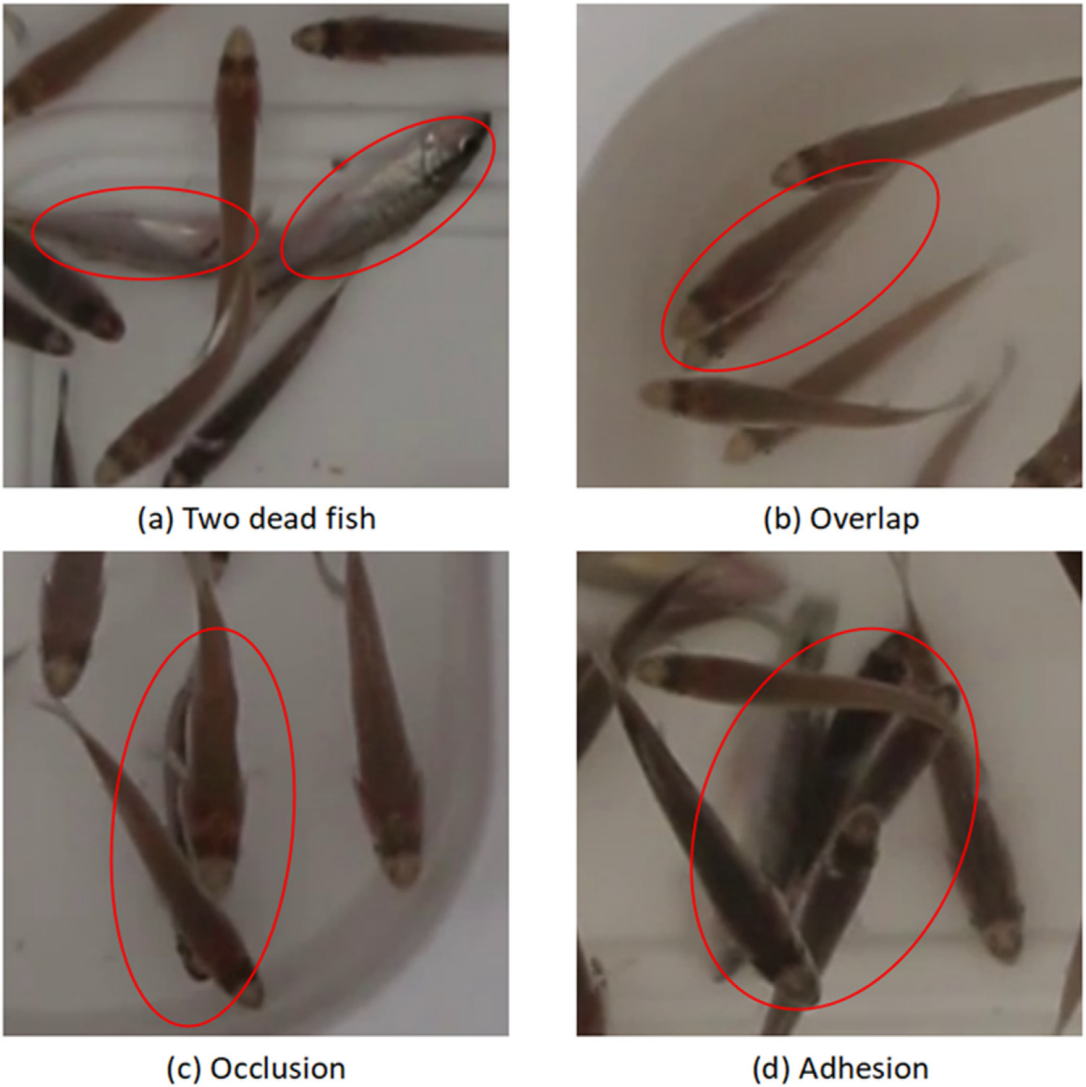
Example of local enlarged image in complex scene.

To collect images suitable for assessing the health of the fish population, varying numbers of dead fish were randomly added to the fish tank during data collection. All dead fish were sourced from the farm on the day of filming. Due to the limited area of the tank, increased fish fry density led to complex scenarios that posed challenges to target detection tasks, including fish overlap, occlusion, and adhesion. Fig. 5 provides examples of dead fish images and three types of complex scenarios in the enlarged views of images from the dataset of this paper. Fig. 5(a) highlights a scenario where two dead fish are present. It is observed that deceased fish typically exhibit a turned-over posture, with whitish bodies. In the dataset, each image contains several dead fish in different states of death, either sunk to the bottom or floating in the water. Throughout the data collection, oxygen was supplied to the fish tank to ensure the welfare of the fish during filming intervals for each density group. The entire collection was conducted under conditions that did not interfere with normal growth and no fish were harmed by stress. Fig. 5(b) demonstrates a scenario of fish overlap, where two fish within the red circle are in close proximity, causing parts of their bodies to coincide within the field of view. This situation commonly occurs in dense schools of fish or during swimming. Fig. 5(c) depicts occlusion within fish schools, where one fish is largely obscured by two others, hindering visibility of the obscured fish. Fig. 5(d) illustrates adhesion among fish schools, where several fish are closely associated for various reasons (e.g., feeding, disease, water quality).

### 4.2 Data labeling

**Fig. 6:**
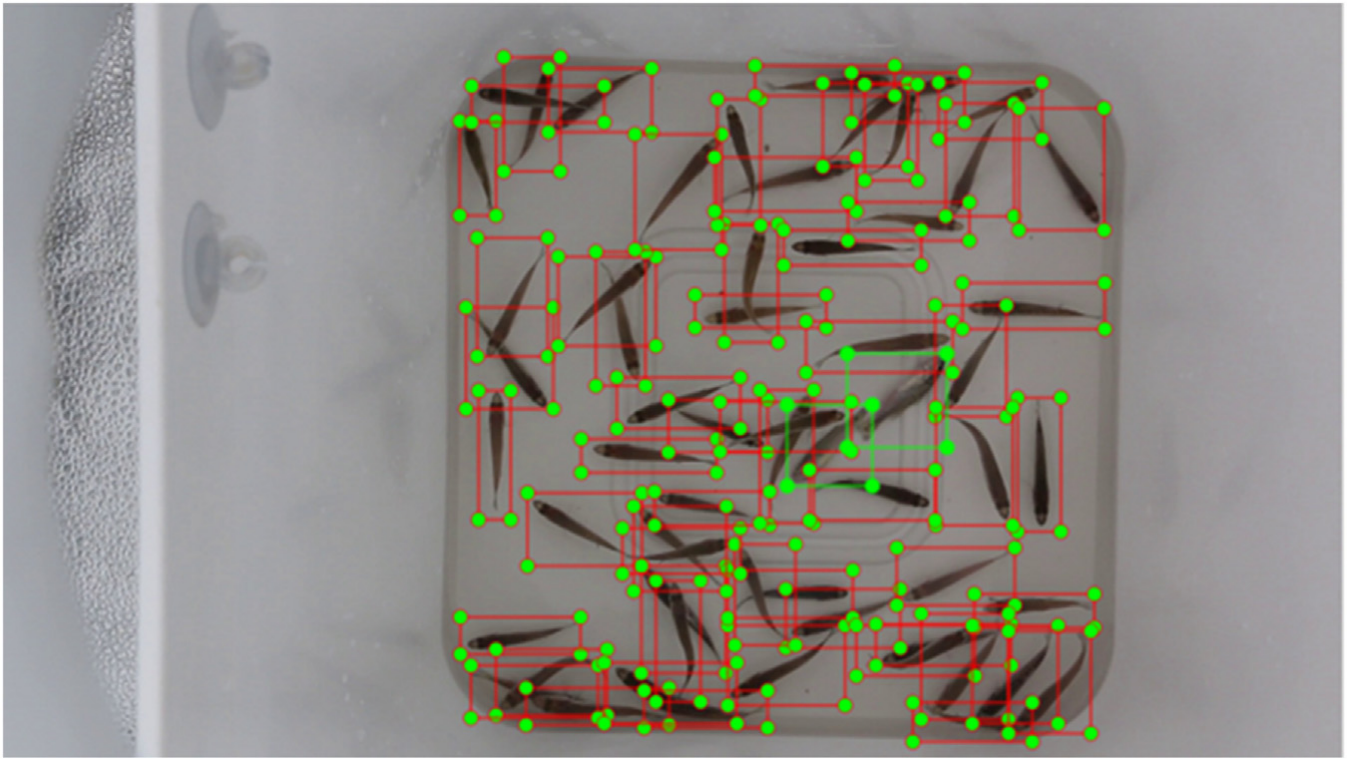
Annotation image at 60 fish density.

Labelimg was employed for image annotation, specifically to identify and label the fish fry within each image using the YOLO format. This process resulted in the generation of corresponding label files in TXT format for each image. Fig. 6 presents annotations at a density of 60 fish, with live fry marked in red and dead fry marked in green. A total of 54,705 fish fry were annotated, consisting of 51,119 specimens and 3596 deceased specimens. To facilitate training on various models, we subsequently converted the YOLO format label files into JSON annotations compatible with the MS COCO framework.

### 4.3 Basic experiments on the dataset

To validate the effectiveness of FFD, we implemented five state-of-the-art deep learning methods: three two-stage detectors (Faster-RCNN [9], VFNet [10] and Cascade-RCNN) [11] and two one-stage detectors (PAA [12] and YOLOv7 [13]) for the fry detection task. In the detection study, we emphasized accuracy, error rate, speed, and model size. The evaluation metrics selected included precision, recall, mean Average Precision at IoU 0.5 (mAP0.5), model size, GFLOPs, and frames per second (FPS). The results of the model performance evaluation metrics are presented in Table 1.

**Table 1:** Performance of different models on the test set.

Algorithm	Precision (%)	Recall (%)	mAP0.5 (%)	Model size (MB)	GFLOPs	FPS
YOLOv7	0.967	0.940	0.977	139.18	103.2	46.4
Cascade-RCNN	0.951	0.969	0.951	68.93	234.47	24.9
Faster-RCNN	0.946	0.964	0.946	41.13	206.67	35.7
PAA	0.960	0.988	0.960	31.89	201.46	11.6
VFNet	0.862	0.956	0.859	32.49	189.02	31.3

**Fig. 7:**
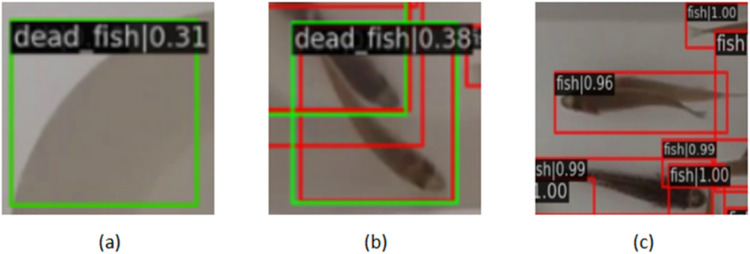
Examples of problematic detection: (a) false alarm, (b) error detection, (c) missed detection.

Subsequently, we trained various models on the dataset and compared the best-performing models across different fry densities and lighting conditions using the test set. The results indicated that in high-density scenarios, certain models exhibited instances of missed detections and false alarms. For instance, both PAA and VFNet mistakenly identified some live fry as dead fry, while Cascade and Faster R-CNN exhibited missed detections in cases of fry overlap and adhesion. The YOLOv7 algorithm demonstrated the best overall performance, effectively addressing detection challenges such as high fry numbers, perspective distortion, and severe overlap. Fig. 7 displays some problematic detection examples, where (a) misidentifies the edge of the fish tank as a dead fish, (b) incorrectly detects a live fish as deceased, and (c) represents a missed detection of a fish in the center of the frame. Consequently, there remains significant scope for improving detection performance relevant to researchers utilizing this dataset for future algorithm optimization and design.

### 4.4 Comparison with existing fish detection datasets

**Fig. 8:**
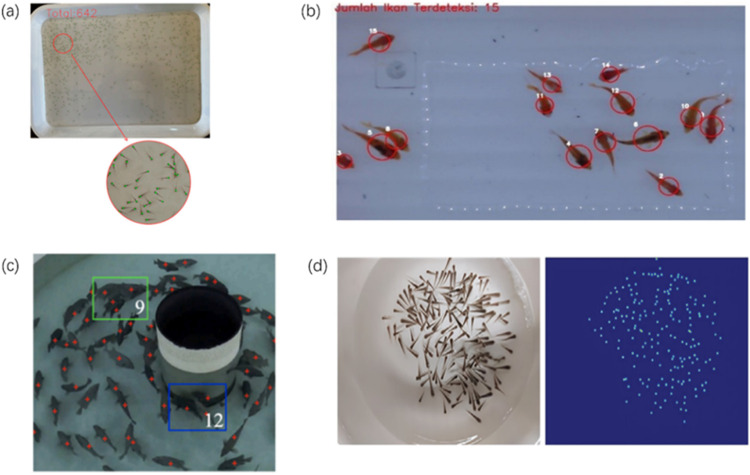
Image examples from existing datasets: (a) [14]; (b) [15]; (c) [16]; (d) [17].

It is noteworthy that fish detection datasets have been published in previous studies, such as [[14], [15], [16], [17]]. The image examples of these datasets are shown in Fig. 8.

Compared with existing datasets, FFD has the following novel contributions.

• The length of the fishes varies in datasets. In Fig. 8(a), (b), and (d), the average length ranges from 1 to 3 cm. For Fig. 8(c), the average length reaches 20 cm. In FFD, the average length is 3–5 cm, which differs from existing datasets.
• Current datasets only consider live fishes in the dataset. However, in FFD, both live and dead fishes were included because this setting was more in line with real aquacutural industry.
• Compared with existing datasets, we had more diverse environmental settings, such as water depth and illumination conditions.
• We provide the popular types of annotations in YOLO and JSON formats. For Fig. 8(a), (c), and (d), these datasets only provided point annotation, which was not suitable for training anchor-based object detection algorithms like Faster-RCNN. The point annotation was only applicable for generating density maps of the image. For Fig. 8(b), this dataset was designed for conventional image processing algorithms by thresholding, therefore, this dataset was not suitable for training deep learning based object detection algorithms.
• Last but not the least, only one dataset (i.e., FishFry-2023 [14]) is publicly available to the research society with a limited dataset size, 200 images in total. On the contrary, the FFD dataset contains more than 1000 images, which provide diverse image samples.

## Limitations

Not applicable.

## Ethics Statement

All procedures in this study were conducted according to the “Guiding Principles in the Care and Use of Animals'' (China) and approved by the Experimental Animal Ethics Committee of Nanjing Agricultural University (NJAULLSC2024032)*.*

## CRediT Author Statement

**Yuqiang Wu:** Methodology, Visualization, Writing, review & editing; **Huanliang Xu:** Conceptualization, Supervision; **Bowen Liao, Jia Nie, Chengxi Xu, Ziao Zhang:** Data curation, Validation; **Zhaoyu Zhai:** Supervision, funding acquisition, review & editing.

## Data Availability

Mendeley DataFish Fry Dataset (Original data).
